# Supplementary data for the mediation effect of personality functioning – Gender differences, separate analyses of depression and anxiety symptoms and inferential statistics of the relationship between personality functioning and different types of child maltreatment

**DOI:** 10.1016/j.dib.2022.108272

**Published:** 2022-05-16

**Authors:** Anna Freier, Johannes Kruse, Bjarne Schmalbach, Sandra Zara, Samuel Werner, Elmar Brähler, Jörg M. Fegert, Hanna Kampling

**Affiliations:** a Department of Psychosomatic Medicine and Psychotherapy, Phillips University Marburg, Marburg, Germany; b Department of Psychosomatic Medicine and Psychotherapy, Justus Liebig University Giessen, Giessen, Germany; c Medical Psychology and Medical Sociology, University Medical Center of the Johannes Gutenberg University Mainz, Mainz, Germany; d Department of Child and Adolescent Psychiatry/Psychotherapy, University of Ulm, Ulm, Germany; e Department Psychosomatic Medicine and Psychotherapy, Johannes Gutenberg University Mainz, Mainz, Germany; f Integrated Research and Treatment Center for Adiposity Diseases, Behavioral Medicine Research Unit, University Medical Center Leipzig, Germany

**Keywords:** Personality functioning, Child maltreatment, Depression, Anxiety, Operationalized Psychodynamic Diagnosis (OPD), Gender differences, Mental health

## Abstract

This article provides supplementary data for the original research article ‘The Mediation Effect of Personality Functioning Between Different Types of Child Maltreatment and the Development of Depression/Anxiety Symptoms – a German Representative Study’ [1]. The representative data were acquired as part of a broader survey on different aspects of mental health in the German population. This supplementary data article contains the statistical analyses of the relationship between the different subtypes of child maltreatment (CM) and impairments of personality functioning, which were only presented as a diagram in the original article. Additionally, the analyses conducted separately for symptoms of depression and anxiety as well as gender are provided. For the mediation analyses, the variables of depression/anxiety symptoms, personality functioning and CM types were standardized. The data may be useful for discussion, further insights and as a starting point for the development of further studies.

## Specifications Table

**Table:** 

Subject	Psychiatry and Mental Health
Specific subject area	*Epidemiological study of the general population regarding mental health outcomes*
Type of data	table, figures
How the data were acquired	Data were acquired in 2016 by a demography consulting company (USUMA, Berlin, Germany) as part of a broader survey on different aspects of mental health. Random route procedure was used to obtain a representative sample of the German population. A study assistant visited all participants at home, informed them about the investigation, and the participants provided written informed consent. Before the survey was conducted by paper and pencil, the research staff obtained the sociodemographic information in an interview format. English versions of the used questionnaires (CTQ, PHQ-4, and OPD-SQS) can be accessed in the repository.
Data format	Raw and analyzed data
Description of data collection	The sample was representative with regard to age, gender, education, and the geographic region of the participants. Inclusion criteria were a minimum age of 14 and sufficient knowledge of the German language; responses were given anonymously.
Data source location	Institution: University of Giessen City/Town/Region: Giessen Country: Germany
Data accessibility	Repository name: Mendeley Data Data identification number: 10.17632/95yysn7cns.1 Direct URL to data: http://dx.doi.org/10.17632/95yysn7cns.1
Related research article	A. Freier et al., “The Mediation Effect of Personality Functioning Between Different Types of Child Maltreatment and the Development of Depression/Anxiety Symptoms – a German Representative Study,” *Journal of Affective Disorders 299 (2022) 408–415*. https://doi.org/10.1016/j.jad.2021.12.020

## Value of the Data

• The data provide results from the statistical analyses regarding the relationship between the different subtypes of child maltreatment (CM), namely emotional, physical and sexual abuse as well as emotional and physical neglect, and impairments of personality functioning
• The data presents the detailed results of the separate mediation analysis of depression and anxiety symptoms as well as gender-specific results of this mediation effect
• Researchers with further interest in the mediating role of personality functioning in the association of CM and depression/anxiety symptoms as well as in gender differences will benefit from the additionally analyzed data
• Researchers can use the detailed information such as the presented differences in depression and anxiety symptoms as well as gender differences, both of which may be useful for discussion, and further insights for the development of further studies

## 1 Data Description

Table 1 shows the ANOVA and *t*-test results of the relationship between personality functioning and the severity degrees of the different subtypes of CM; the descriptive statistics and the bar graphs of this relationship are presented in the original article [1]. Most of the differences between the severity degrees of the different CM subtypes were significant. The largest difference was found in the CM subtype sexual abuse between the moderate and severe severity. In cases of emotional abuse, emotional neglect and physical neglect, there were no significant differences between moderate and severe CM severity regarding the impairments of personality functioning. For physical abuse and sexual abuse, the differences between minimal and moderate severity were not significant.

**Table 1:** ANOVA and t-Test between the severity degrees for each type of child maltreatment (CM).

CTQ severity			none vs. minimal		minimal vs. moderate		moderate vs severe	
CTQ subscale	ANOVA	*p*	*t*-Test	*p*	*t*-Test	*p*	*t*-Test	*p*
emotional abuse n = 2310	*F*(3,2306)=156.42	≤.001*	*t*(339.66 ^a^)=−11.96	≤.001*	*t*(361)=−4.55	≤.001*	*t*(137)=−0.63	*.*531
physical abuse n = 2320	*F*(3, 2316)= 58.23	≤*.*001*	*t*(2172)=−5.42	≤.001*	*t*(205)=−1.89	.060	*t*(139.10^a^)=−2.51	.013*
sexual abuse n = 2322	*F*(3,2318)= 91.01	≤.001*	*t*(2147)=−8.34	≤.001*	*t*(259)=−0.010	.992	*t*(171)=−5.94	≤.001*
emotional neglect n = 2319	*F*(3,2315)= 51.09	≤.001*	*t*(1068.03^a^)=−8.27	≤.001*	*t*(773)=−3.25	.001*	*t*(296.99 ^a^)=0.84	.401
physical neglect n = 2307	*F*(3,2303)= 22.07	≤.001*	*t*(1779)=−2.35	.019*	*t*(758)=−2.64	.008*	*t*(524)=−1.94	.053

*Note.* *p < 0.05

.^a^*degree of freedom adjusted based on Levene's test for equality of variances.*

OPD-SQS = Operationalized Psychodynamic Diagnosis - Structure Questionnaire Short. CTQ = Childhood Trauma Questionnaire.

**Fig. 1:**
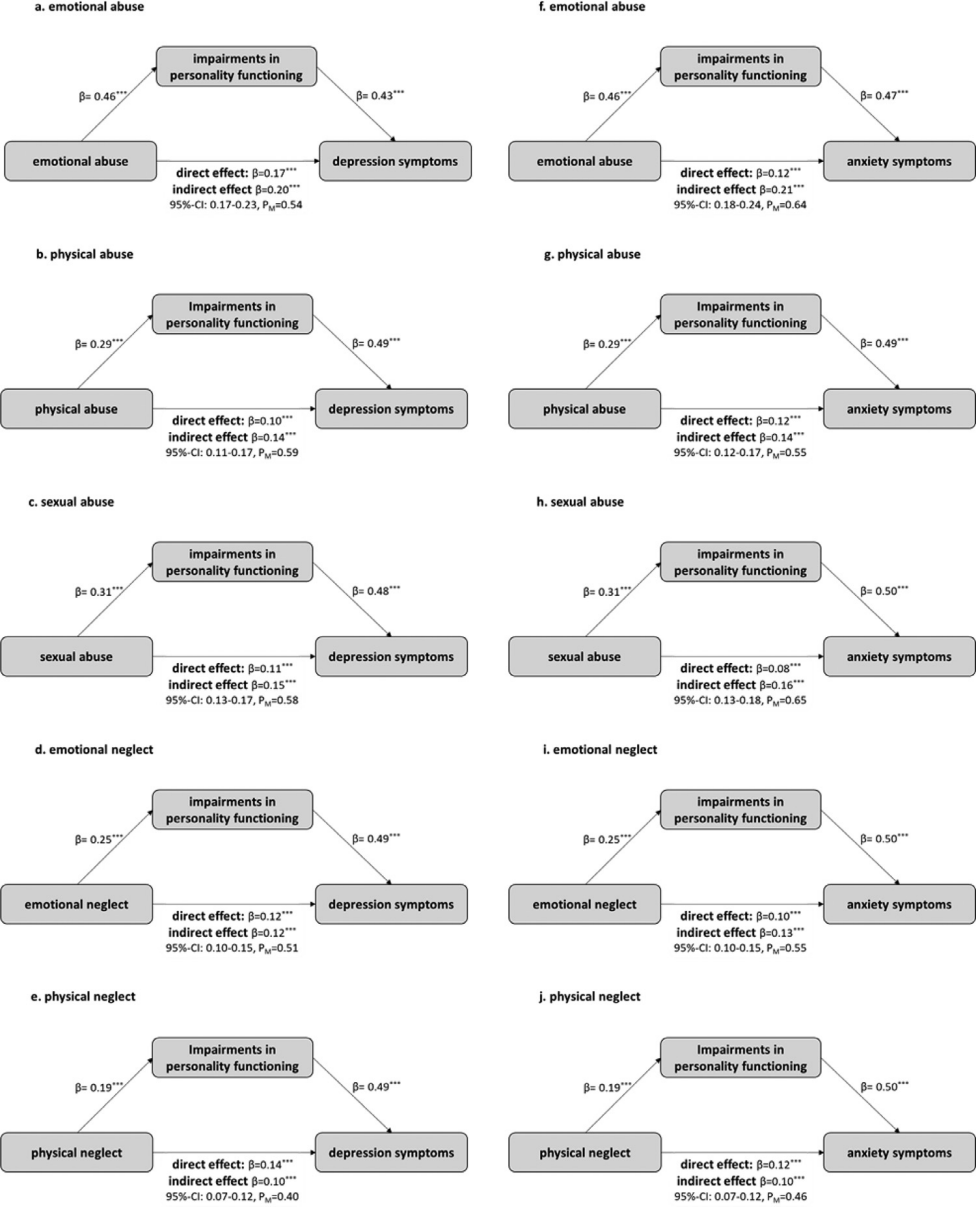
Mediating models of personality functioning in the relationship between CM and depression (a.-e.) and anxiety (f.-j.) symptoms. Standardized β-coefficients are reported.

*note.* *p < 0.05; ^⁎⁎^*p* < 0.01; ^⁎⁎⁎^*p* < 0.001; CI = confidence interval; P_M_ = proportion of the mediating effect as proportion of the total effect.

Fig. 1 presents the diagrams of the mediation models for the separate analysis of depression and anxiety symptoms. The results are in a comparable range and are all significant. The largest difference was found in the CM subtype emotional abuse, with the mediating (indirect) effect for anxiety symptoms being stronger (64%) than for depression symptoms (54%).

**Fig. 2:**
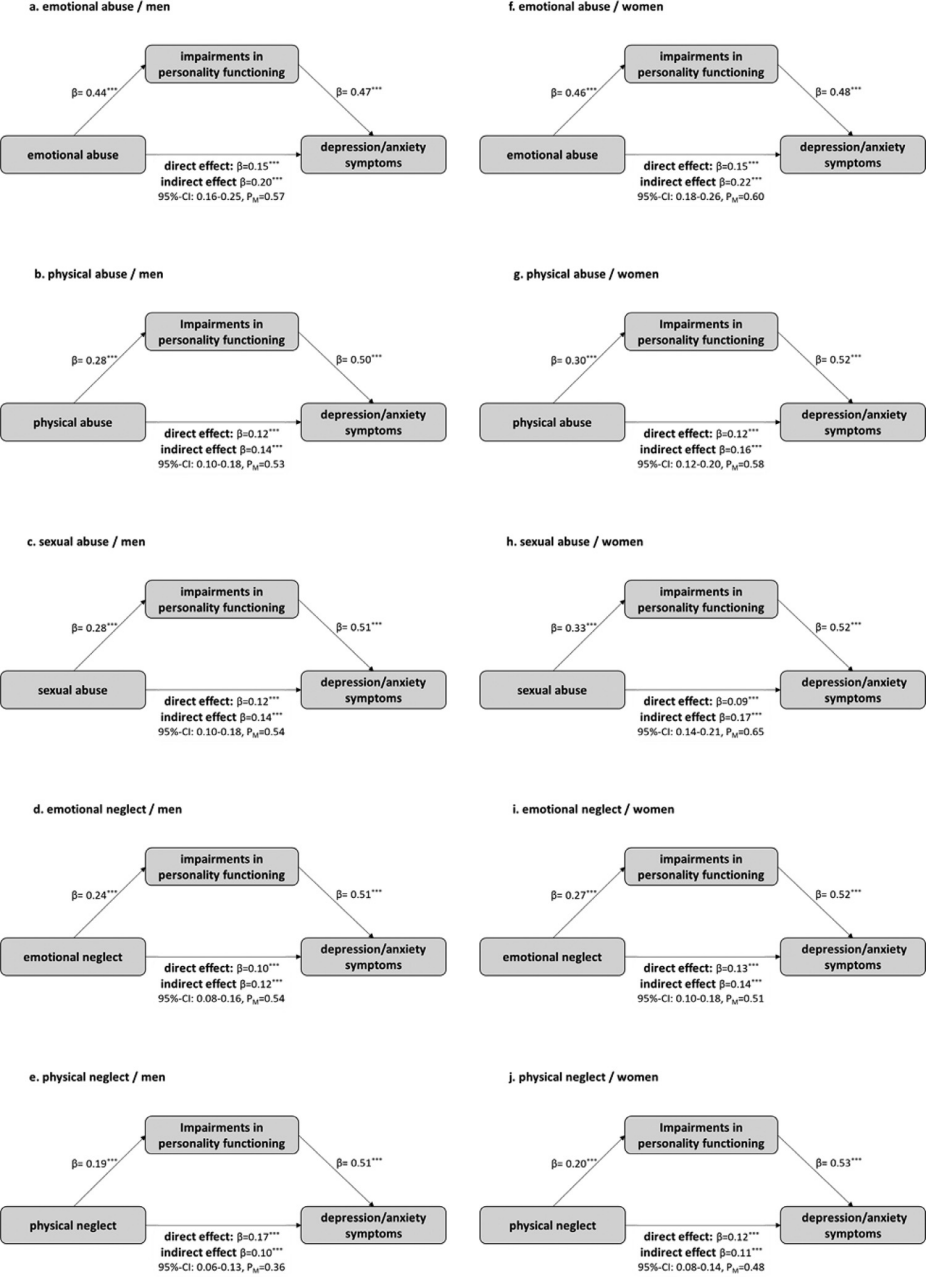
Gender-specific mediating models of personality functioning in the relationship between CM and depression/anxiety symptoms. Diagrams a.-e. present the mediating models for men and f.-j. for women. Standardized β-coefficients are reported.

*note.* *p < 0.05; ^⁎⁎^*p* < 0.01; ^⁎⁎⁎^*p* < 0.001; CI = confidence interval; P_M_ = proportion of the mediating effect as proportion of the total effect.

Fig. 2 depicts the diagrams of the gender-specific analyses of the mediation effect of personality functioning between different CM subtypes and depression/anxiety symptoms. The mediating effects were similar for all subtypes of CM and the results were all significant. The largest difference was found in the CM subtype physical neglect, where the proportion of the mediating effect was less for men (36%) than for women (48%). A similarly large difference was found in the CM subtype sexual abuse, with the mediating effect for women being stronger (65%) than for men (54%).

An Excel file (‘Raw_Data_AFreier_MediationPersonalityFunctioning.xlsx’) contains the raw data for all variables as well as the filter and sum scores of the used questionnaires.

## 2 Experimental Design, Materials and Methods

### 2.1 Participants

The full study sample included *N* = 2510 participants. For the reason that personality functioning can be considered as a developmental process, *n* = 86 underage participants who are yet developing personality functioning were excluded. Primary analyses require complete data for both OPD-SQS and PHQ-4. Therefore, participants with missing values in either of these questionnaires were excluded. In case of the CTQ, participants were excluded only from analyses on specific CM types if there were missing values, resulting in specific sample sizes for each CM type: emotional abuse *n* = 2310, physical abuse *n* = 2320, sexual abuse *n* = 2322, emotional neglect *n* = 2319, and physical neglect *n* = 2307.

### 2.2 Procedure

The study data was acquired in cooperation with an independent demography consulting company (USUMA, Berlin, Germany) as part of a broader survey on different aspects of mental health between September and November 2016. Random-route procedure was used to select households in all regions of Germany (based on electoral districts) to obtain a representative sample of the German population. Only participants who were at least 14 years of age and had sufficient German language skills were included. Initially, *N* = 4838 households were contacted. For multi-person households, one person was randomly selected by means of the Kish selection grid technique. *n* = 2324 participants were excluded for different reasons (e.g., they refused to take part, were not at home or on vacation, due to illness; for more details see [1]). A total of *N* = 2510 participants were interviewed successfully. A study assistant visited these participants at home and informed them about the investigation and the anonymity of their answers. Then the participants provided written informed consent. In the next step, the study assistant obtained the socio-demographic information in form of face-to-face interview. The survey was conducted via paper and pencil. If the participant needed more information about questions of the survey, the study assistant offered support. However, the study assistant was not involved in the completion of the survey. Finally, the socio-demographic data of the face-to-face interview and the survey were linked foregoing personal data like name and address.

### 2.3 Materials

During the interview socio-demographic information (age, gender, level of education and personal income) was collected. All other questionnaires used are available in English in the Mendeley data link repository.

The types of CM were measured with the Childhood Trauma Questionnaire (CTQ) ([2], German version: [3]), which is a self-report screening questionnaire distinguishing five subtypes of CM: emotional, physical and sexual abuse, as well as emotional and physical neglect. Each subtype of CM was assessed with five items, which were rated on a five-point Likert-type scale with response options ranging from 1 = “never true” to 5 = “very often true”. Moreover, the CTQ has three items for detecting underreporting of maltreatment, a minimization/denial validity scale (e.g., “I had the perfect childhood”). Internal consistency was weakest for physical neglect (α=0.55); all other subtypes ranged between α=0.80 (physical abuse) and α=0.89 (sexual abuse). Häuser et al. [4] suggest severity degrees for each subscale based on norm data. They postulate threshold scores to determine the severity of each subscale in the following degrees: “none”, “minimal”, “moderate”, and “severe”. In the case of our study, we chose the cut-off criterion “moderate” for the prevalence calculation for the different types of CM.

Symptoms of depression and anxiety were measured by the Four-Item Patient Health Questionnaire (PHQ-4) [5]. The PHQ-4 is a brief tool which includes the PHQ-2 screening tool for depression and the GAD-2 screener for generalized anxiety disorders (GAD). According to the Diagnostic and Statistical Manual of Mental Disorders (DSM-IV; [6]), PHQ-2 assesses self-report of two core symptoms of depression and GAD-2 collects two core symptoms of general anxiety disorder with a view to the past two weeks. It uses a four-point Likert scale ranging from 0 = “not at all” to 3 = “nearly every day”; the total score ranges from 0 to 12. The PHQ-4 has shown acceptable reliability with an internal consistency of α = 0.78 in the general population [7].

For the assessment of personality functioning, a short version of the OPD Structure Questionnaire (OPD-SQS; [8]) was used. The OPD-SQS comprises 12 items which are rated on a five-point Likert scale from 0 = “does not apply at all” to 4 = “fully applies”. The sum score ranges from 0 to 48, with higher scores indicating impairments in personality functioning. Moreover, three subscales (self-perception, interpersonal contact, and relationship model), which are highly intercorrelated, were computed. The subscale “self-perception” contains aspects of the self with structural skills of emotion regulation. The subscale “relationship model” characterizes the representation of experiences in relationships and the expectations of new relationships. The subscale “interpersonal contact” represents the interpersonal skills combined with aspects of self-uncertainty. However, in this study only the total score was used. A recent study [9] reported good reliability and validity of the OPD-SQS for the general population, independent of gender and age.

### 2.4 Statistical analysis

In accordance with prior analyses, supplementary analyses were conducted in R (Version 4.0.2; [10]). Descriptive statistics of the relationship between CM subtypes and personality functioning on which the ANOVAs were based, can be found in the original research article [1]. First, ANOVAs for each CM subscale were calculated. Then, if the ANOVA results were significant, post-hoc *t*-tests between the degrees of CM severity were conducted in a secondary step. If the Levene's test for equality of variances was significant, the degree of freedom was adjusted.

For all mediation analyses, the R package lavaan [11] was used. For the calculation of the separate mediation analyses of depression and anxiety symptoms, the two items for depression (PHQ-2) and the two items for anxiety (GAD-2) were added in separate variables. Subsequently, all required variables were standardized. For the gender-specific mediation analyses the data file was split in separate samples.

In the mediation analysis, the total effect describes the association between each of the CM types and depression/anxiety symptoms. This total effect is the additive result from two components: the direct and the indirect effect. The direct effect refers to the part of the total effect that occurs without the mediating variable, and the indirect effect refers to the part of the total effect that is the result of mediation alone. For a better understanding, the proportion of the mediating (indirect) effect as a proportion of the total effect was also calculated [12, 13]. Mediation emerges when the indirect effect is significant and the confidence intervals exclude zero [14].

## Ethics Statements

The study was approved by the Ethics Committee of the Medical Department of the University of Leipzig (ref: 297/16-ek). The study was conducted in accordance with the Declaration of Helsinki and fulfilled the ethical guidelines of the International Code of Marketing and Social Research Practice of the International Chamber of Commerce, and of the European Society of Opinion and Marketing Research. All participants provided informed written consent. In the case of underage participants, informed consent was also obtained from a parent/legal guardian.

## CRediT Author Statements

**Anna Freier:** conceptualization, formal analysis, methodology, writing - original draft; **Johannes Kruse:** project administration, conceptualization, data curation, supervising, writing - review & editing; **Bjarne Schmalbach:** methodology, writing - review & editing; **Sandra Zara:** writing - review & editing; **Samuel Werner:** writing - review & editing; **Elmar Brähler:** data curation, writing - review & editing; **Joerg M. Fegert:** data curation, writing - review & editing; **Hanna Kampling:** project administration, conceptualization, supervising, writing - review & editing.

## Declaration of Competing Interest

The authors declare that they have no known competing financial interests or personal relationships that could have appeared to influence the work reported in this paper.

## Data Availability

Supplementary Data for the Mediation Effect of Personality Functioning (Original data) (Mendeley Data).
